# Chronic intermittent hypoxia exacerbates isoproterenol-induced cardiac hypertrophy and apoptosis

**DOI:** 10.3389/fcvm.2025.1700967

**Published:** 2026-01-06

**Authors:** Yujie Zhang, Ming Zhang, Hongfeng Jiang, Fang Fang

**Affiliations:** 1 Experimental Research Center, Beijing Institute of Heart Lung and Blood Vessel Disease, Beijing Anzhen Hospital, Capital Medical University, Beijing, China; 2 Department of Cardiology, Beijing Anzhen Hospital, Capital Medical University, Beijing, China; 3 Department of Structural Heart Disease, National Center for Cardiovascular Disease & Fuwai Hospital, Chinese Academy of Medical Sciences and Peking Union Medical College, Beijing, China; 4 National Health Commission Key Laboratory of Cardiovascular Regeneration Medicine, Beijing, China

**Keywords:** chronic intermittent hypoxia, isoproterenol, cardiomyocyte hypertrophy, cardiac remodeling, apoptosis, PI3K/Akt/mTOR pathway

## Abstract

**Background:**

Obstructive sleep apnea (OSA) is marked by chronic intermittent hypoxia (CIH) and iassociated with multiple cardiovascular complications. Isoproterenol (ISO) is commonly used to induce cardiac hypertrophy. However, the impact of CIH on ISO-induced cardiac hypertrophy and remodeling remains unclear.

**Methods:**

Cardiac hypertrophy was induced in mice using ISO, with or without CIH. Echocardiography was performed to assess cardiac functions, while histological analyses were employed to evaluate the physiological modifications in the heart. Western blotting and real-time quantitative PCR were used to evaluate protein and mRNA gene expression levels, respectively. Additionally, immunofluorescence was employed to observe the morphological changes in H9C2 cells.

**Results:**

CIH exacerbated ISO-induced cardiac dysfunction and cardiac pathological alterations in mice. The expression of atrial natriuretic peptide (ANP) and brain natriuretic peptide (BNP) was elevated in both mice and H9C2 cells in the CIH + ISO group. Furthermore, CIH exacerbated ISO-induced cell apoptosis. We additionally observed that the PI3K/AKT/mTOR pathway is further activated by the co-induction of CIH and ISO.

**Conclusions:**

CIH exhibits a negative effect on ISO-treated mice and cells, leading to an exacerbation of cardiac dysfunction and remodeling. In addition, CIH aggravates ISO-treated cardiomyocyte apoptosis in H9C2 cells.

## Introduction

Pathological cardiac hypertrophy is characterized by the enhancement of cardiomyocytes, reconfiguration of cardiac structure, and elevated expression of atrial natriuretic peptide (ANP) and brain natriuretic peptide (BNP) (1, 2). A variety of pathological states, such as hypertension, obesity, and cardiomyopathy, can lead to cardiac hypertrophy (3). The progression of cardiac hypertrophy can lead to a state of decompensation, characterized by reduced contractile function and deteriorating cardiac performance, ultimately culminating in heart failure (4). Therefore, it is crucial to ascertain effective treatments and comprehend the underlying mechanisms that prevent the development of cardiac hypertrophy.

Obstructive sleep apnea (OSA) is the most prevalent sleep disorder characterized by recurrent episodes of either total or partial upper airway obstruction during sleep, leading to chronic intermittent hypoxia (CIH) and hypercapnia (5). Among middle-aged individuals, OSA is diagnosed in approximately 34% of men and 17% of women (6). OSA can contribute to the progression or exacerbation of cardiovascular diseases, with prevalence rates ranging from 40% to 80% in patients with hypertension, coronary artery disease, heart failure, and atrial fibrillation (7). Notably, OSA has been acknowledged as an independent risk factor for various cardiovascular diseases, and it increases all-cause incidence (6, 8).

CIH is a significant pathology characterized by frequent upper airway collapse during sleep, linking OSA and cardiac dysfunction (9). A growing number of studies indicate that CIH exposure induces myocardial damage and elevates the mortality rates in heart failure in animal models (10). CIH significantly influences the progression of cardiac hypertrophy, potentially due to oxidative stress (11) and systemic inflammation (12). Nevertheless, the additional factors underlying OSA-associated cardiac hypertrophy require further investigation.

In the current study, we investigated the effects of CIH on the development of isoproterenol (ISO)-induced cardiac hypertrophy in mice and H9C2 cardiomyocytes.

## Materials and methods

### Animal model

We purchased C57BL/6 mice (male, aged 8 weeks, weighing 22–25 g) from Beijing Sibeifu Biotechnology Co., Ltd, China. The animals were accommodated in the Experimental Animal Center of Beijing Institute of Heart Lung and Blood Vessel Disease, at 21 ℃–24 ℃ and 40%–60% humidity with a 12 h light/dark cycle and *ad libitum* access to food and water. A total of 32 mice were randomly divided into four groups (*n* = 8 per group). (1) The NC group was subcutaneously injected with saline in a volume equal to the volume of the ISO solution (10 mg/kg/d; Sigma-Aldrich, USA) and housed under normal oxygen conditions for 14 days (13). (2) The ISO group was subcutaneously injected with ISO and continuously maintained in a normoxic environment for 14 days. (3) The CIH group was subcutaneously injected with saline solution and kept under intermittent hypoxia (5% O_2_ at nadir, 20 cycles/h) for 14 days. (4) The CIH + ISO group was subcutaneously injected with ISO and housed under intermittent hypoxia for 14 days. Investigators performing echocardiography, sample collection, and histological and data analysis were blinded to group allocation throughout the study to minimize bias. To induce CIH, the mice were fed in customized standard cages during a 12 h dark phase (OxyCycler A84 system, BioSpherix, USA). The system facilitated precise modulation of the inspired oxygen fraction, ranging from 21% to 5%, over 120 s intervals, followed by swift restoration to a normoxic level of 21% oxygen within the next 60 s. Under the 12 h light phase, the mice were kept under normoxic conditions (14). All animal experiments in the current study were approved (approval number: AZ2024LA007) by the Animal Care and Ethics Committee of Beijing Anzhen Hospital (Beijing, China).

### Echocardiography

The mice were anesthetized with 2% isoflurane. Cardiac performance was assessed and analyzed utilizing the Vevo 2100 ultrasound system (VisualSonics, Canada). M-mode imaging in the parasternal left ventricular short-axis view was employed to assess cardiac function. Basic indices, including left ventricular anterior wall thickness at end systole (LVAWs), left ventricular anterior wall thickness at end diastole (LVAWd), left ventricular posterior wall thickness at end systole (LVPWs), left ventricular posterior wall thickness at end diastole (LVPWd), left ventricular internal diameter at end systole (LVIDs), left ventricular internal diameter at end diastole (LVIDd), ejection fraction (EF), and fractional shortening (FS), were measured (15). Heart rate was monitored during echocardiographic measurements. At least three cardiac cycles were measured each time.

### Histology

After the mice had been sacrificed using intraperitoneal injection of 1% pentobarbital sodium (60 mg/kg), the cardiac tissues were quickly removed and weighed. Myocardial tissues were fixed, embedded in paraffin, and sectioned into 5 µm slices. Next, these sections were stained with hematoxylin and eosin (HE), Masson's trichrome, and wheat germ agglutinin (WGA) (1:200, Sigma-Aldrich, L4895) for morphometric analysis, as well as immunofluorescence staining to assess capillary density using CD31 (1:500, Abcam, ab182981) and arteriolar density using α-SMA (1:1,000, Proteintech Group, 67735-1-ig) (16). Images were captured using a fluorescence microscope (Nikon ECLIPSE Ni, Nikon, Japan). Quantitative analysis of cell surface area, collagen content, and fluorescence intensity was performed using the ImageJ software (NIH, USA).

### Cell model

H9C2 rat cardiomyocytes were purchased from Wuhan Pricella Biotechnology Co., Ltd., China, and cultured in DMEM medium (Gibco, USA) at 37 °C in a 5% CO_2_ incubator. Cells were plated in culture dishes at approximately 80% confluence to perform the experiments. To create experimental hypertrophy, they were exposed to 50 μM ISO for 48 h (17). CIH was induced in H9C2 cells using a customized hypoxia chamber at 37 °C with 5% CO_2_. The oxygen level was cyclically varied between 21% O_2_ for 25 min and 5% O_2_ for 35 min through controlled N_2_ infusion (OxyCycler C42, BioSpherix) (18).

### Determination of cell surface area

Cultured H9C2 cells were fixed with 4% fresh paraformaldehyde solution and permeabilized with 0.5% Triton X-100. The cells were then washed thrice with PBS and blocked with 2% bovine serum albumin for 1 h (19). Subsequently, they were incubated with α-actin (1:50 dilution, Immunoway, YT0097) for 1 h to illustrate the structure of actin filaments. The cells were further stained with Hoechst (Sigma-Aldrich), and the surface area was assessed using a fluorescence microscope.

### Cell viability assay

The Cell Counting Kit 8 (CCK8) assay was performed to assess cell viability (20). The cells were seeded in 96-well culture plates and placed in an incubator for 48 h. Subsequently, CCK8 reagent was added (10 + 90 μL complete medium) to each well. The absorbance value (450 nm) was measured using a microplate reader after incubation for 2 h.

### TUNEL assay

Apoptosis of cardiomyocytes was assessed using the TUNEL assay with a commercial kit (C1062, Beyotime, China). Following cell fixation and permeabilization, Annexin V-FITC was added for staining in the dark for 20 min. Subsequently, the proportion of cells exhibiting green fluorescence was determined using a fluorescence microscope.

### RNA extraction and real-time qPCR

Total RNA was extracted from cultured H9C2 cells using TRIzol reagent (Sigma-Aldrich), following the protocol provided by the manufacturer. To synthesize cDNA, RNA was subjected to reverse transcription using an M-MLV Kit (Invitrogen, USA). Quantitative real-time PCR was conducted to determine gene expression levels, utilizing PowerUp SYBR Green Master Mix (Invitrogen). GAPDH served as a normalization control. The relative expression levels of mRNA were calculated using the 2-ΔΔCt method. The primer sequences utilized are detailed in Supplementary Table 1.

### Western blotting

H9C2 cells or mouse cardiac tissues were homogenized in RIPA lysis buffer (Solarbio, China). The protein concentration was measured using a BCA Protein Assay Kit (Beyotime, China). Protein samples (20 μg) were separated using 12% SDS-PAGE and transferred onto PVDF membranes (Millipore, USA). Membranes were blocked with 5% non-fat dry milk for 1 h and incubated with primary antibodies at 4 °C overnight. The following antibodies were used: ANP (1:1,000, Abcam, ab189921), BNP (1:1,000, Thermo Fisher Scientific, PA5-102596), caspase-3 (1:500, Thermo Fisher Scientific, 43-7800), cleaved caspase-3 (1:1,000, Cell Signaling Technology, 9664), Bax (1:2,000, Abmart, T40051), Bcl-2 (1:500, Abmart, T40056), PI3K (1:1,000, Affinity Biosciences, AF6241), p-PI3K (1:1,000, Affinity Biosciences, AF3241), Akt (1:1,000, Cell Signaling Technology, 4,691), p-Akt (1:1,000, Cell Signaling Technology, 9271), mTOR (1:1,000, Cell Signaling Technology, 2983), p-mTOR (1:1,000, Cell Signaling Technology, 5536), and GAPDH (1:10,000, Abcam, ab181602). After incubation with secondary antibodies, goat anti-rabbit IgG (1:20,000, ZSGB-Bio, ZB-5301) or goat anti-mouse IgG (1:20,000, ZSGB-Bio, ZB-5305) for 1 h, protein blots were imaged using the Quantity One software (Bio-Rad, USA). GAPDH was employed as an endogenous gene. Band signal intensities were analyzed using the ImageJ software.

### Statistical analysis

Data are presented as the mean ± standard error of the mean (SEM). An unpaired two-tailed Student's *t*-test was used for data comparison between two groups, and one- or two-way analysis of variance (ANOVA) was used for multiple group comparisons (21). Statistical significance was set at *P* < 0.05. GraphPad Prism 8 (GraphPad Software, USA) was used for statistical analysis.

## Results

### CIH exacerbates ISO-induced cardiac dysfunction in mice

**Figure 1:**
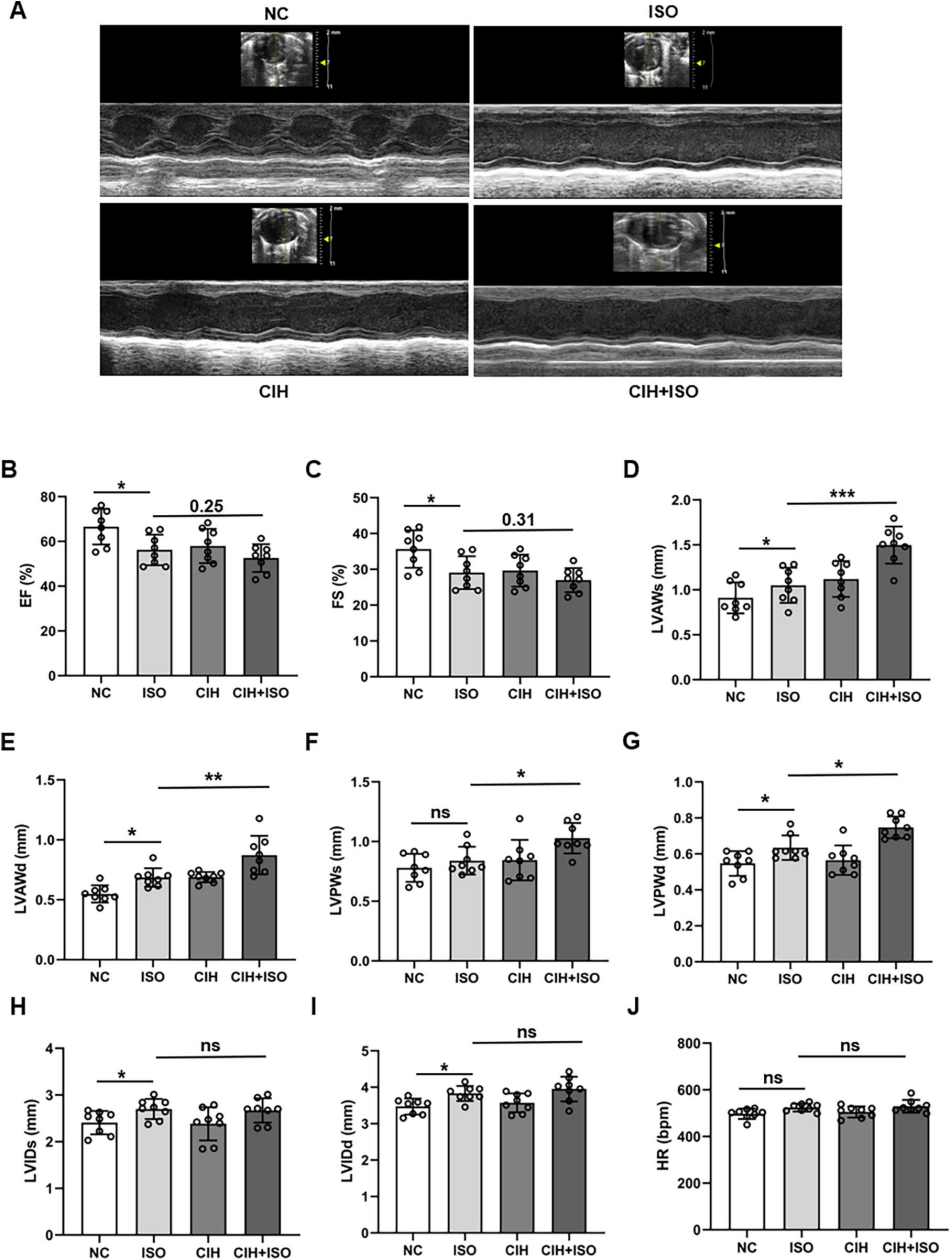
CIH worsens ISO-treated cardiac dysfunction in mice. **(A)** Representative left ventricular echocardiography images in different groups. **(B–J)** Changes in EF, FS, LVAWs, LVAWd, LVPWs, LVPWd, LVIDs, LVIDd, and heart rate in the different groups. Data are expressed as the mean ± SEM (*n* = 8). *P < 0.05, **P < 0.01, and ***P < 0.001. ns, no significant difference.

To determine whether CIH aggravates ISO-induced cardiac hypertrophy, echocardiographic analysis was performed among the four groups (NC, ISO, CIH, and CIH + ISO). The echocardiography results revealed that EF and FS were reduced in the ISO group and further reduced under CIH conditions, although there was no statistical significance compared with the ISO group (Figures 1A–C). ISO treatment increased the thickness of the anterior and posterior walls of the left ventricle during both diastole and systole, and these effects were further exacerbated under CIH-inducing conditions (Figures 1D–G). However, combined treatment with CIH and ISO increased both LVIDs and LVIDd, but there was no statistical difference compared with the levels in the ISO group (Figures 1H,I). The heart rate in the ISO and CIH + ISO groups was slightly increased, albeit not statistically significant (Figure 1J). These data demonstrate that CIH exposure exacerbates cardiac dysfunction in ISO-treated mice.

### CIH aggravates ISO-induced cardiac pathological remodeling in mice

**Figure 2:**
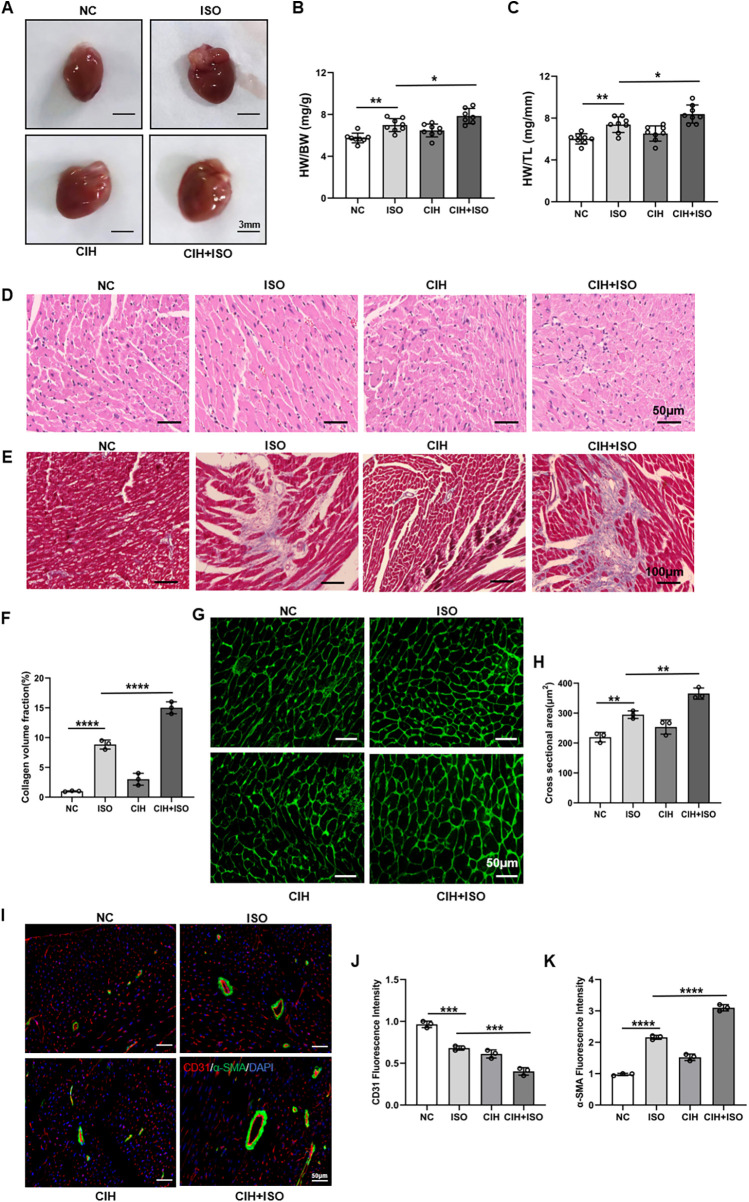
CIH aggravates cardiac histopathological remodeling and cardiomyocyte hypertrophy in ISO-treated mice. **(A)** Gross morphology of the whole heart. **(B)** HW/BW and **(C)** HW/TL ratios. **(D)** HE staining of cardiac sections. **(E)** Masson's trichrome staining of cardiac tissue. **(F)** Bar graph showing changes in collagen deposition and myocardial fibrosis. **(G)** WGA staining of cardiac tissue. **(H)** Bar graph showing changes in cardiomyocyte cross-sectional areas. **(I)** CD31 (red), α-SMA (green), and DAPI (blue) immunofluorescence staining. **(J,K)** Bar graph showing changes in fluorescence intensity of CD31 and *α*-SMA. Data are expressed as the mean ± SEM (*n* = 8). *P < 0.05, **P < 0.01, ***P < 0.001, and ****P < 0.0001.

As shown in Figure 2A, the mice cardiac dimensions were considerably increased in the ISO-treated group, which were further enlarged in the CIH + ISO group. Furthermore, heart weight to body weight (HW/BW) and heart weight to tibia length (HW/TL) ratios were elevated in ISO-treated mice, with increased values in mice exposed to CIH-inducing conditions (Figures 2B,C). To explore the contribution of CIH to ISO-induced cardiac hypertrophy and fibrosis, histopathological analysis of cardiac tissues by HE and Masson's trichrome staining revealed that ISO treatment markedly exacerbated pathological cardiac remodeling, characterized by excessive collagen deposition and myocardial fibrosis (Figures 2D–F). These abnormalities were markedly worsened in mice exposed to a CIH-inducing environment. The dimensions of cardiomyocyte cross-sections were determined using WGA staining. Our results showed that the ISO group exhibited an enlargement in the surface area of cardiomyocytes, with CIH further exacerbating this increase (Figures 2G,H). Quantitative immunofluorescence analysis of CD31 and α-SMA demonstrated that ISO treatment considerably increased α-SMA expression levels and decreased CD31 levels in cardiac tissues, whereas these alterations were further amplified under CIH conditions (Figures 2I–K). These results demonstrate considerably increased vascular remodeling in the CIH + ISO group. Therefore, our data illustrate that CIH aggravates ISO-induced cardiac and vascular pathological remodeling as well as myocardial fibrosis and hypertrophy in mice.

### CIH worsens ISO-induced cardiac hypertrophy both *in vitro* and *in vivo*

**Figure 3:**
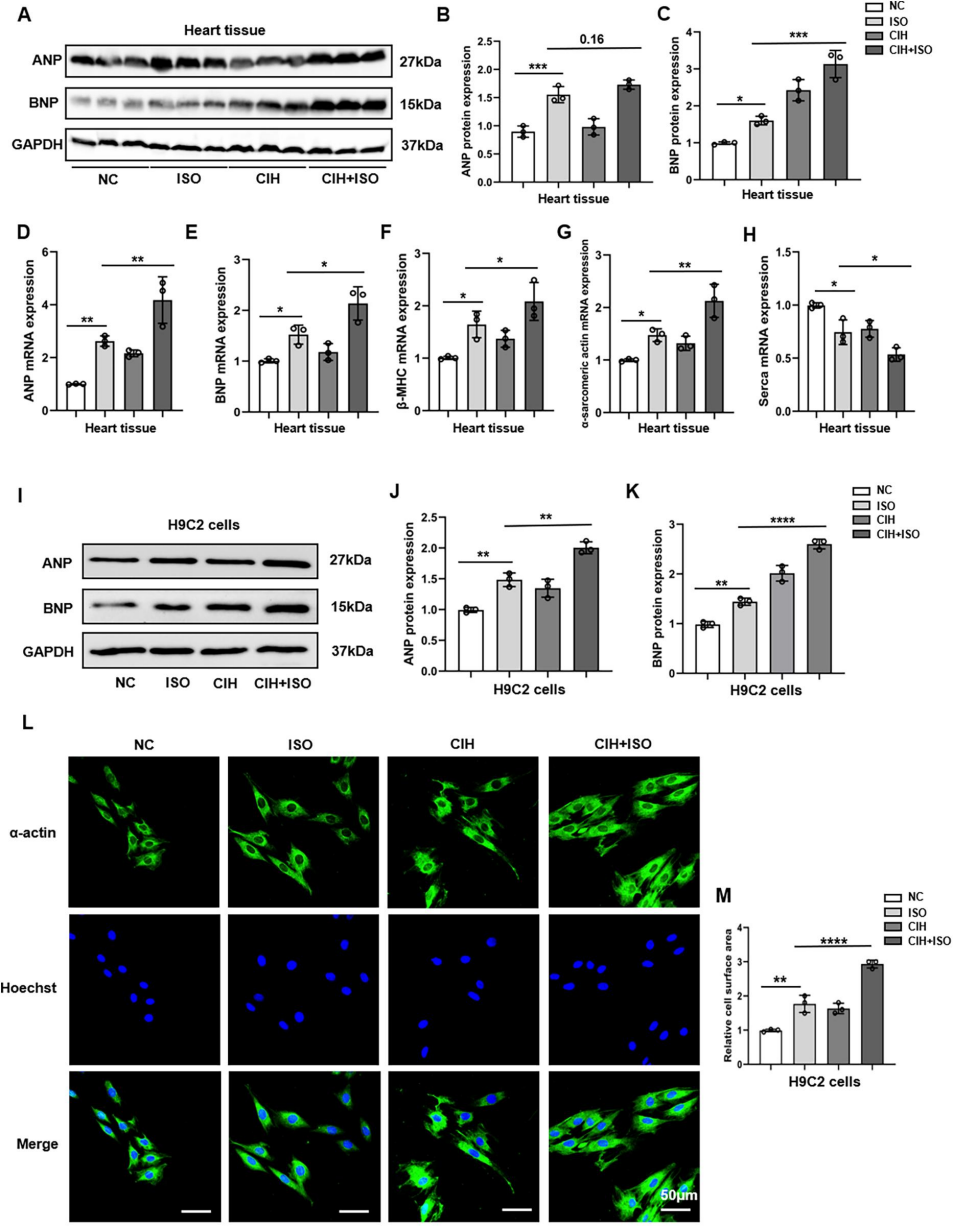
CIH exacerbates cardiomyocyte hypertrophy induced by ISO *in vitro* and *in vivo*. **(A–C)** Western blot and quantitative analyses of ANP and BNP expression in mouse heart tissues. **(D–H)** mRNA levels of ANP, BNP, β-MHC, α-sarcomeric actin, and SERCA were detected through qPCR in mouse heart tissues. **(I–K)** The protein levels of ANP and BNP were measured in H9C2 cells using Western blotting. **(L)** Surface area of H9C2 cells. **(M)** Bar graph showing changes in H9C2 cross-sectional areas. Data are expressed as the mean ± SEM (*n* = 3). *P < 0.05, **P < 0.01, ***P < 0.001, and ****P < 0.0001.

We measured ANP and BNP protein levels using Western blotting and observed an increase in both levels within the cardiac tissues of mice that were treated with ISO, which further escalated after CIH treatment (Figures 3A–C). We also measured the mRNA expression levels of cardiac hypertrophy markers, including ANP, BNP, β-myosin heavy chain (β-MHC), α-sarcomeric actin, and sarcoplasmic/endoplasmic reticulum calcium ATPase (SERCA), and observed remarkable upregulation of ANP, BNP, β-MHC, and α-sarcomeric actin, as well as downregulation of SERCA (Figures 3D–H).

Based on the robust induction of ANP and BNP expression (Supplementary Figures 1A–D), a regimen of 50 μM ISO for 48 h was established to model cardiomyocyte hypertrophy in H9C2 cells for subsequent investigations. Our results indicated that the protein and mRNA levels of ANP and BNP notably increased in the CIH + ISO group compared with those in the ISO and CIH groups (Figures 3I–K; Supplementary Figures 2A–E). The surface area of H9C2 cardiomyocytes markedly increased after ISO treatment and was further enlarged following CIH induction (Figures 3L,M). In conclusion, our findings reveal that CIH worsened ISO-induced cardiomyocyte hypertrophy in both mice and H9C2 cells.

### CIH augments the effect of ISO-induced cell apoptosis

**Figure 4:**
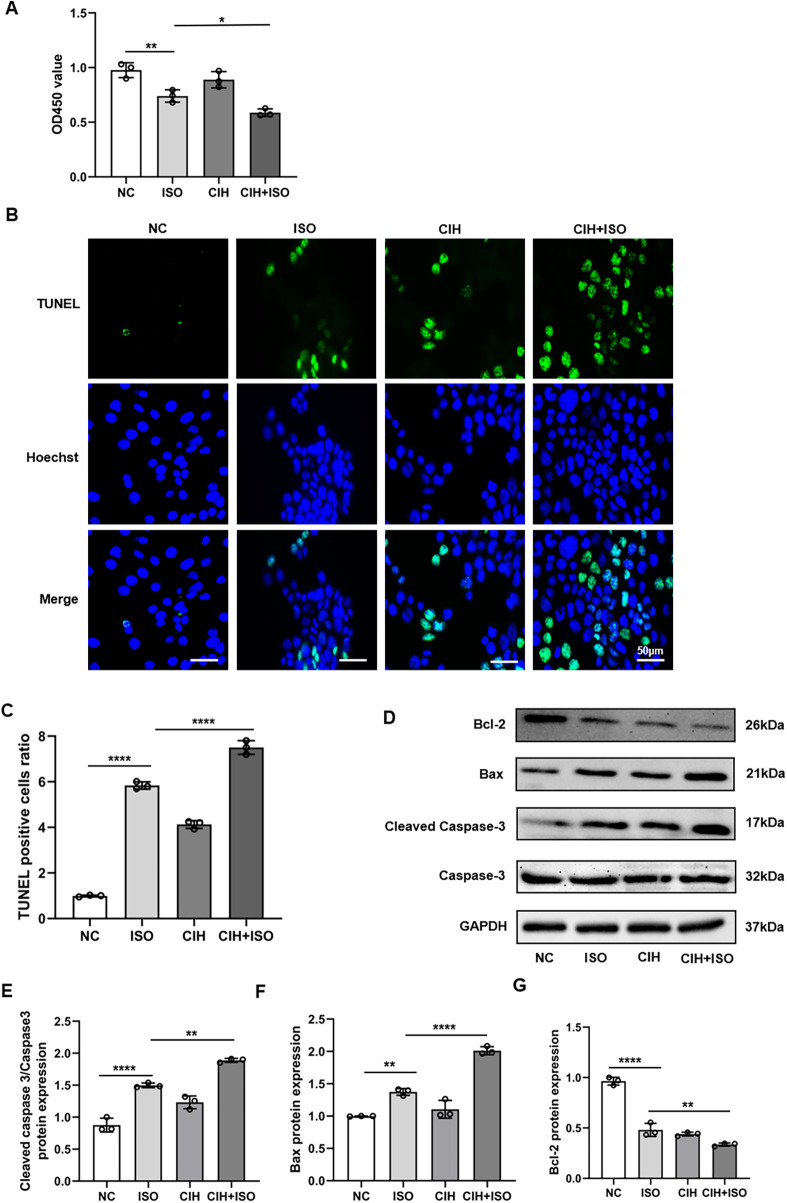
CIH aggravates ISO-induced apoptosis in cardiomyocytes. **(A)** CCK8 assay of viable cells. **(B)** TUNEL (green) and Hoechst (blue) double staining and **(C)** quantification of TUNEL + cardiomyocytes. **(D–G)** Western blot and quantitative analyses of cleaved caspase-3, caspase-3, Bax, and Bcl-2 expression. Data are expressed as the mean ± SEM (*n* = 3). *P < 0.05, **P < 0.01, and ****P < 0.0001.

ISO treatment for 48 h significantly induced apoptosis in H9C2 cardiomyocytes. The CCK8 assay results showed that OD450 values were lower in the ISO group than those in the NC group, whereas the values in the CIH + ISO group were further decreased compared with those in the ISO group (Figure 4A). The degree of apoptosis was further assessed using the TUNEL assay. The results indicated that the number of TUNEL-positive cells was considerably higher in the CIH + ISO group compared with those in the ISO and CIH groups, suggesting an enhanced apoptotic response in the CIH + ISO condition (Figures 4B,C). Additionally, cleaved caspase-3/caspase-3 and Bax protein levels increased, whereas Bcl-2 expression decreased in the CIH + ISO group, suggesting that CIH might promote cardiomyocyte apoptosis (Figures 4D–G). Taken together, these results demonstrate that CIH enhanced the influence of ISO on cell apoptosis.

### Impact of CIH and ISO on the PI3K/Akt/mTOR pathway

**Figure 5:**
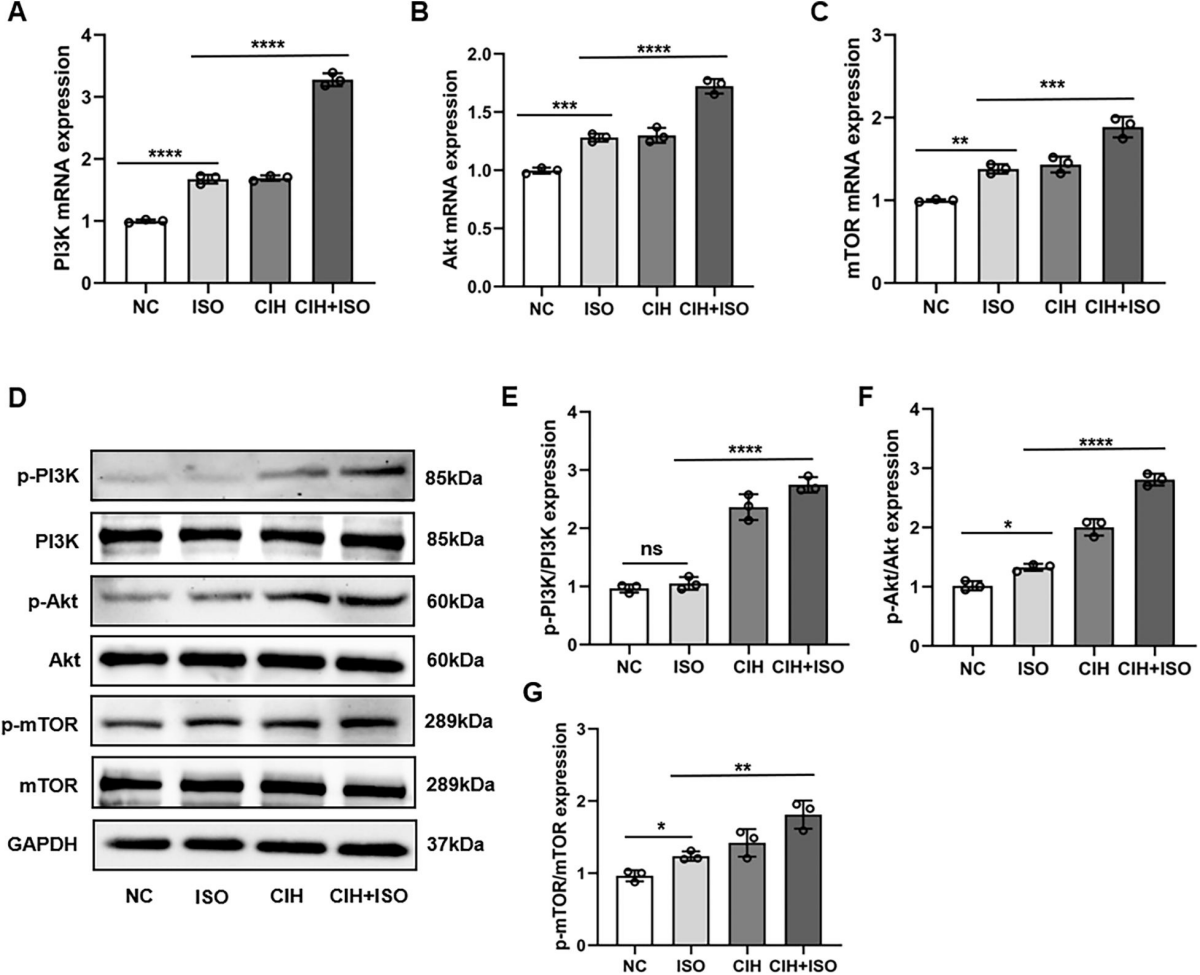
Effect of CIH and ISO on the PI3K/Akt/mTOR pathway in cardiomyocytes. **(A–C)** mRNA levels of PI3K, Akt, and mTOR were detected through qPCR. **(D–G)** Western blot and quantitative analyses of p-PI3K, PI3K, p-Akt, Akt, p-mTOR, and mTOR expression. Data are expressed as the mean ± SEM (*n* = 3). *P < 0.05, **P < 0.01, ***P < 0.001, and ****P < 0.0001. ns, no significant difference.

Previous studies have demonstrated that the PI3K/Akt/mTOR signaling pathway plays a crucial role in regulating cardiac hypertrophy. To investigate the roles of the PI3K/AKT/mTOR signaling pathway in regulating CIH and ISO-induced hypertrophy, we measured the RNA and protein levels in cardiomyocytes. The RNA expression of PI3K, Akt, and mTOR was markedly increased in the CIH + ISO group (Figures 5A–C). Additionally, we observed a considerable increase in the protein levels of p-PI3K/PI3K, p-Akt/Akt, and p-mTOR/mTOR induced by CIH and ISO (Figures 5D–G).

## Discussion

Cardiac hypertrophy is considered an adaptive response that occurs in response to mechanical and neurohumoral stimuli. Prolonged cardiac hypertrophy leads to serious cardiac abnormalities, ultimately resulting in heart failure (22). ISO has been widely used to establish cardiac hypertrophy in experimental models (23, 24). Therefore, we treated mice with ISO to establish a mouse model of cardiac hypertrophy, while those treated with saline solution served as controls. We found that CIH aggravated pathological cardiac hypertrophy in ISO-treated mice, characterized by worsened EF, thickened ventricular wall, and deteriorated cardiac and vascular pathological remodeling. Furthermore, we found that cardiomyocyte apoptosis induced by ISO was exacerbated in the CIH induction group.

Clinical epidemiological data indicate that OSA is associated with cardiovascular disease (25, 26). Further studies have shown that OSA can significantly amplify the risk of cardiovascular events, leading to cardiac hypertrophy and heart failure (27–30). Treatment of OSA primarily involves the application of continuous positive airway pressure (CPAP) during sleep. However, the adherence to CPAP therapy among patients with OSA significantly varies, which may limit treatment efficacy (31, 32). In a retrospective case–control study involving 32 patients with OSA and 19 patients without OSA, left ventricular remodeling was elevated in OSA, showing a preserved left ventricular ejection fraction with cellular hypertrophy (33). A meta-analysis showed that CPAP treatment improved the performance of both left and right ventricles in patients with OSA (34). An earlier clinical trial indicated that in patients with heart failure who were on medical therapy, additional CPAP treatment for OSA could improve left ventricular systolic function (35). Additionally, a recent randomized controlled trial showed that the concurrent use of CPAP therapy in patients with heart failure and OSA can significantly reduce pulmonary arterial hypertension and improve biventricular function (36). However, a meta-analysis suggested that CPAP treatment does not have a significant impact on the left ventricular EF in patients with OSA. Moreover, adherence to CPAP treatment greatly varies among individuals with OSA, which limits therapeutic outcomes (37).

OSA exerts adverse effects on cardiovascular physiology through multiple intricate mechanisms, with CIH being recognized as the primary pathogenic feature that promotes and exacerbates cardiac pathological remodeling and cardiac injury (38). Our study confirmed the effects of CIH on ISO-induced cardiac hypertrophy in mouse models. We observed that cardiac functions such as left ventricular EF and FS declined, whereas left ventricular anterior and posterior wall thickness increased when ISO-induced mice were exposed to CIH. Additionally, concurrent exposure to ISO and CIH (CIH + ISO) caused marked morphological alterations, including an enlarged heart, increased HW/BW and HW/TL ratios, and pronounced enhancement of cardiac remodeling. This pathological progression was characterized by upregulated expression of hypertrophy biomarkers (ANP, BNP, β-MHC, and α-sarcomeric actin), exacerbated cardiomyocyte hypertrophy with sarcomere disarray, aggravated fibrosis evidenced by increased collagen deposition (Masson's trichrome staining), and increased vascular remodeling (CD31 and α-SMA immunofluorescence staining). Our results demonstrate that CIH exposure considerably exacerbates ISO-induced pathological cardiac and vascular remodeling. Therefore, it is crucial to recognize the effect of potential modulators that mediate CIH-induced cardiac hypertrophy and damage.

Apoptosis is a series of molecular events that ultimately result in cell death. It is recognized as a major pathogenic mechanism that links OSA and cardiac hypertrophy (39). Some studies have indicated that CIH increases the expression of cleaved caspase-3 and Bax (40), which promotes cardiomyocyte apoptosis and exacerbates left ventricular remodeling. Therefore, inhibiting apoptosis could potentially serve as a protective strategy to mitigate myocardial injury (41). Additionally, an experiment on mice showed that an integrator of the apoptosis signaling pathway drove CIH-induced cardiac hypertrophy (42). In this study, we observed that treatment with ISO considerably reduced cell viability and increased the number of TUNEL-positive cells, as well as elevated levels of cleaved caspase-3/caspase-3 and Bax. These effects were further exacerbated by CIH induction. Therefore, CIH may further facilitate the progression from adaptive hypertrophy to heart failure in cardiomyocytes that are already injured by ISO treatment. Additional preclinical studies are warranted to elucidate the precise pathways that connect CIH with the development of heart failure.

Previous studies have shown that CIH activates the PI3K/Akt/mTOR pathway, a critical regulator of cellular survival and hypertrophy (43). Consistent with these findings, our data demonstrated remarkable upregulation of p-PI3K, p-AKT, and p-mTOR protein levels in CIH-treated cells. Notably, ISO co-treatment further enhanced this activation, suggesting that the combined effects of CIH and ISO may drive more severe pathological cardiac remodeling through amplified PI3K/Akt/mTOR signaling.

Our study has some limitations. Our results indicate that CIH potentially aggravates ISO-induced cardiomyocyte hypertrophy and death through enhanced activation of the PI3K/Akt/mTOR signaling pathway. Nevertheless, further intervention studies are required to validate this hypothesis. Moreover, ISO is a prototypical β-adrenergic receptor (β-AR) agonist used to induce cardiac hypertrophy and apoptosis (44); however, cardiac hypertrophy is a multifaceted condition that involves mechanisms beyond β-AR. The ISO-induced myocardial hypertrophy model is based on animal experiments; therefore, the pathophysiological changes it induces may differ from the natural progression of human myocardial hypertrophy. In addition, the exclusive use of male mice precludes assessment of sex as a biological variable. Accordingly, exploring the effects of CIH in additional cardiac hypertrophy models, such as transverse aortic constriction, is warranted in future research.

In conclusion, our data indicate that CIH exacerbates ISO-induced cardiac dysfunction, cardiomyocyte remodeling, and cardiac hypertrophy in an animal model and apoptosis in H9C2 cells. Our investigation reveals that CIH can exacerbate myocardial injury in mice with impaired cardiac function, offering a new perspective on the potential negative impact of CIH under specific pathological conditions. The PI3K/Akt/mTOR pathway was further activated by CIH and ISO, suggesting its potential role in CIH-induced apoptosis and cardiomyocyte hypertrophy, which warrants further experimental validation.

## Data Availability

The datasets presented in this study can be found in online repositories. The names of the repository/repositories and accession number(s) can be found in the article/Supplementary Material.
