# Supplementary material for: Chronic intermittent hypoxia exacerbates isoproterenol-induced cardiac hypertrophy and apoptosis
Source: Front Cardiovasc Med. 2026 Jan 6;12:1700967. doi: 10.3389/fcvm.2025.1700967 (PMC12815717; doi:10.3389/fcvm.2025.1700967)
Supplement: Supplementary file 1 [file Table1.docx]

**1.Supplementary Figures Legend**

**Supplementary Fig. 1** The mRNA levels of (A) ANP and (B) BNP four different ISO-treated concentrations are detected by qPCR in H9C2 cells. The mRNA levels of (C) ANP and (D) BNP in three different ISO-treated times are detected by qPCR in H9C2 cells. Data are expressed as mean ± SEM (n = 3). **P < 0.01, ***P < 0.001, and ****P < 0.0001; ns, no significant difference.

**Supplementary Fig. 2** The mRNA levels of (A) ANP, (B) BNP, (C) β-MHC, (D) α-sarcomeric actin and (E) Serca in H9C2 cells. Data are expressed as mean ± SEM (n = 3). *P < 0.05, **P < 0.01, ***P < 0.001 and ****P < 0.0001; ns, no significant difference.

**Supplementary Table 1** The primer sequences used for the qRT-PCR.

| **Primers for Rat** | **Forward Sequence (5'-3')** | **Reverse Sequence (5'-3')** |
| --- | --- | --- |
| GAPDH | GAAGGTCGGTGTGAACGGAT | CCCATTTGATGTTAGCGGGAT |
| ANP | GGTACCGAAGATAACAGCCAAATC | GGCCTCACTAAACCACTCATCTA |
| BNP | CAGTCTCCAGAACAATCCACGAT | ATCCGGTCTATCTTCTGCCCAAA |
| β-MHC | TCCTCCCTCAAGCTCCTAAGTAA | GCGCAGGTTTGTCATAAGTTTGT |
| alpha-sarcomeric actin | GACTGTCACCTAGTGCCTGC | CAAAGCTGTGGTCTCCTCGT |
| Serca | CTCATGGACGAGACGCTCAA | CAGACCACCAGGGGCATAAT |
| PI3K | TGCCCCTCCTGATGTTGC | TCGGCGAGATAGCGTTTG |
| Akt | GCTACAAGGAACGGCCTCAG | GTTTCCACATGGAAGGTGCG |
| mTOR | TGGAGAACCAGCCCATAAGAAA | TGAGAGAAATCCCGACCAGTGA |
| **Primers for Mice** | **Forward Sequence (5'-3')** | **Reverse Sequence (5'-3')** |
| GAPDH | ACTCTTCCACCTTCGATGCC | TGGGATAGGGCCTCTCTTGC |
| ANP | GGCTTCTTCCTCGTCTTGG | ATCTGTGTTGGACACCGCA |
| BNP | CGGTCTCAAGGCAGCAC | GTTACAGCCCAAACGACT |
| β-MHC | CCTACGATTATGCGTTCATC | CCTGTCAGCTTGTAAATGGA |
| alpha-sarcomeric actin | GATGGACGGGAAGACAGCTC | GATGGACGGGAAGACAGCTC |
| Serca | CTACCTGGAACAACCCGCAA | TCATGCAGAGGGCTGGTAGA |
