# Supplementary figures and images for: Chronic intermittent hypoxia exacerbates isoproterenol-induced cardiac hypertrophy and apoptosis
Source: Front Cardiovasc Med. 2026 Jan 6;12:1700967. doi: 10.3389/fcvm.2025.1700967 (PMC12815717; doi:10.3389/fcvm.2025.1700967)

## Slide 1
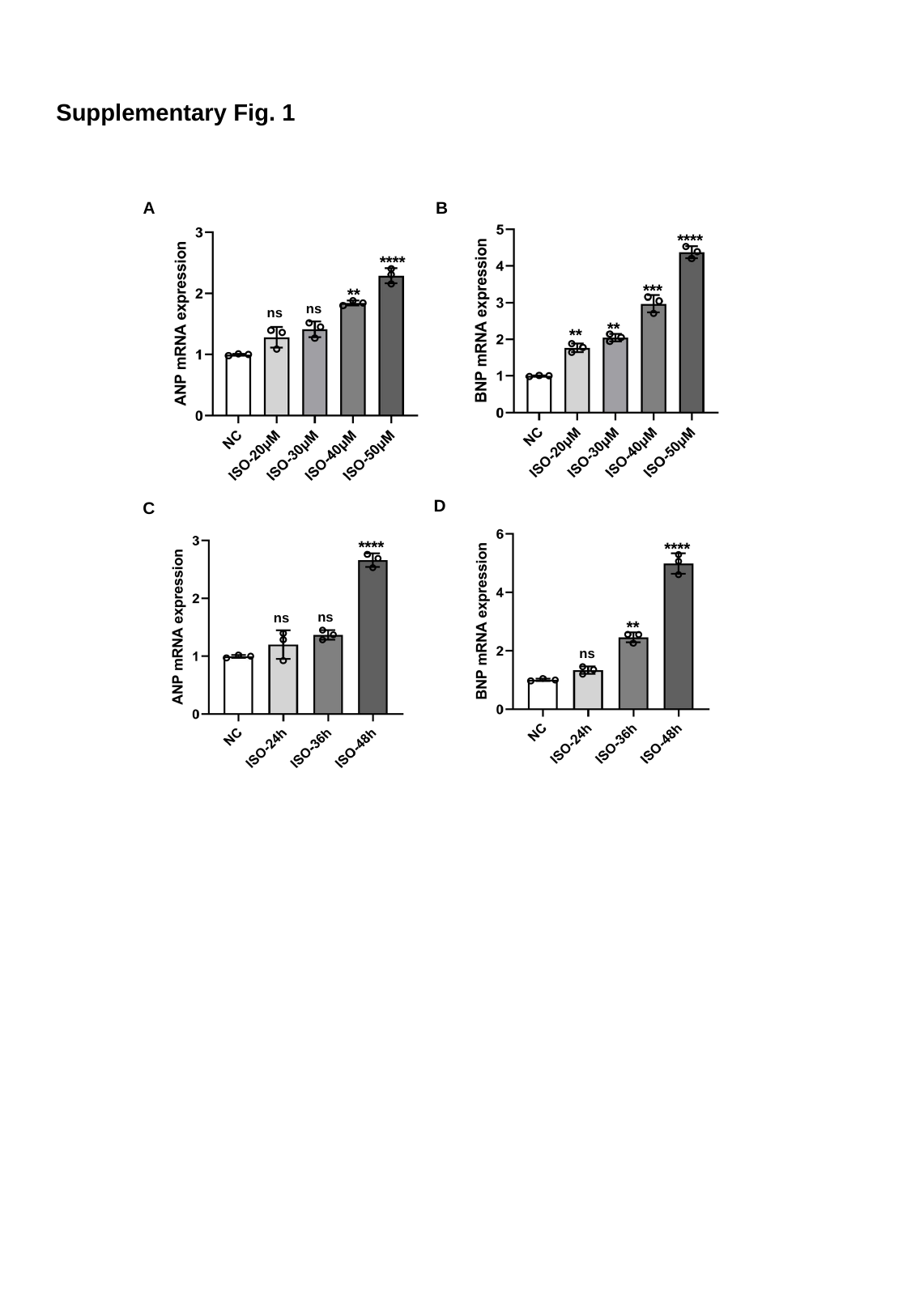

Supplementary Fig. 1
A
B
****
***
**
**
****
**
ns
ns
D
C
****
ns
ns
****
**
ns

## Slide 2
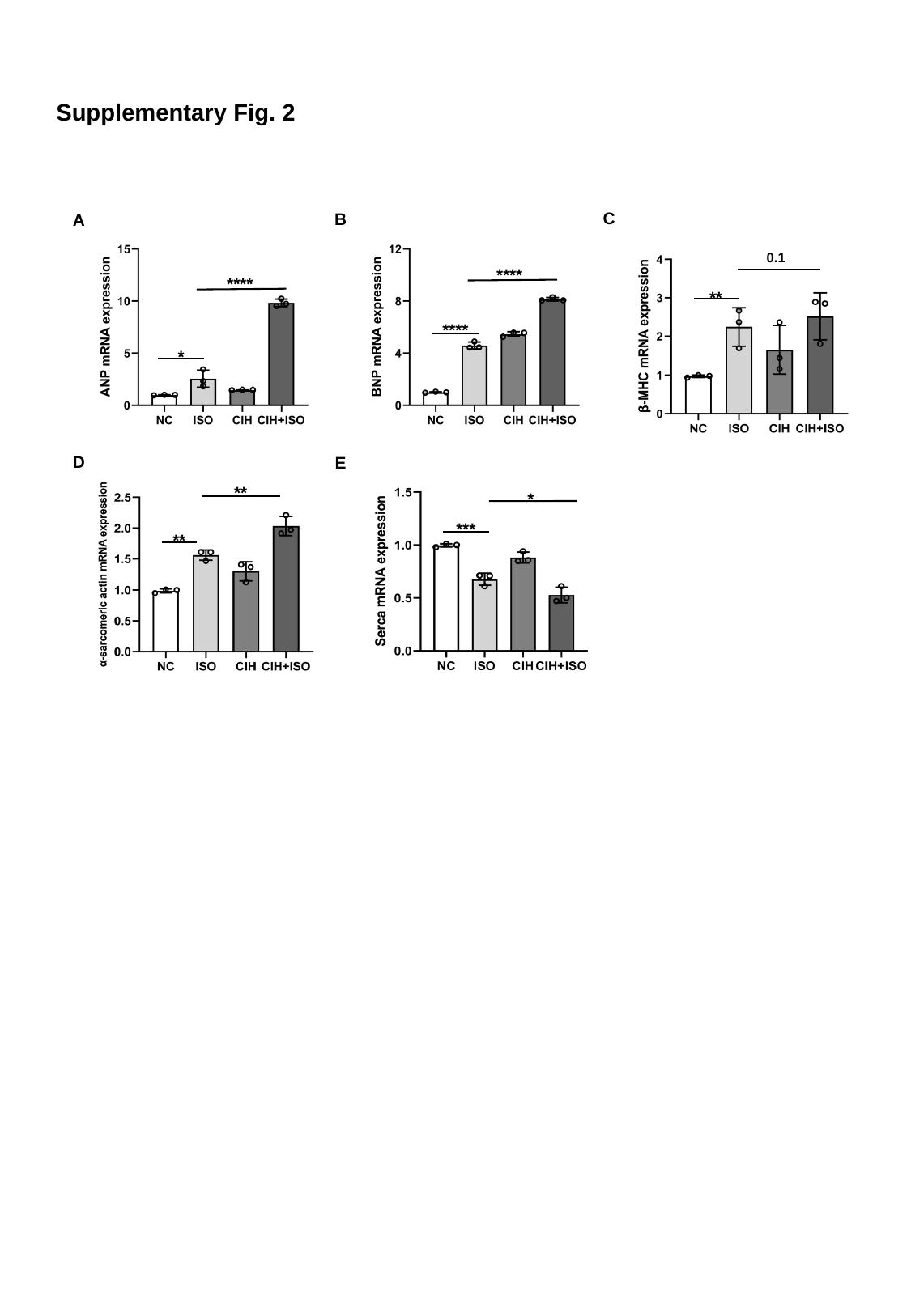

Supplementary Fig. 2
C
B
A
****
*
****
****
0.1
**
D
E
*
***
**
**

Supplement: Supplementary file 2 [file Presentation1.pptx]
